# Small extracellular vesicle DNA-mediated horizontal gene transfer as a driving force for tumor evolution: Facts and riddles

**DOI:** 10.3389/fonc.2022.945376

**Published:** 2022-08-08

**Authors:** Gábor Valcz, Beáta Újvári, Edit I. Buzás, Tibor Krenács, Sándor Spisák, Ágnes Kittel, Zsolt Tulassay, Péter Igaz, István Takács, Béla Molnár

**Affiliations:** ^1^ MTA-SE Molecular Medicine Research Group, Eötvös Loránd Research Network, Budapest, Hungary; ^2^ School of Life and Environmental Sciences, Centre for Integrative Ecology, Deakin University, Waurn Ponds, VIC, Australia; ^3^ Department of Genetics, Cell- and Immunobiology, Semmelweis University, Budapest, Hungary; ^4^ ELKH-SE Immune-Proteogenomics Extracellular Vesicle Research Group, Semmelweis University, Budapest, Hungary; ^5^ HCEMM-SU Extracellular Vesicle Research Group, Semmelweis University, Budapest, Hungary; ^6^ 1st Department of Pathology and Experimental Cancer Research, Semmelweis University, Budapest, Hungary; ^7^ Institute of Enzymology, Research Centre for Natural Sciences, Budapest, Hungary; ^8^ Institute of Experimental Medicine, Eötvös Loránd Research Network, Budapest, Hungary; ^9^ Department of Internal Medicine and Oncology, Semmelweis University, Budapest, Hungary; ^10^ Department of Endocrinology, Semmelweis University, Budapest, Hungary

**Keywords:** extracellular vesicles, exosomes, horizontal gene transfer, tumor evolution, cell-cell communication

## Abstract

The basis of the conventional gene-centric view on tumor evolution is that vertically inherited mutations largely define the properties of tumor cells. In recent years, however, accumulating evidence shows that both the tumor cells and their microenvironment may acquire external, non-vertically inherited genetic properties *via* horizontal gene transfer (HGT), particularly through small extracellular vesicles (sEVs). Many phases of sEV-mediated HGT have been described, such as DNA packaging into small vesicles, their release, uptake by recipient cells, and incorporation of sEV-DNA into the recipient genome to modify the phenotype and properties of cells. Recent techniques in sEV separation, genome sequencing and editing, as well as the identification of new secretion mechanisms, shed light on a number of additional details of this phenomenon. Here, we discuss the key features of this form of gene transfer and make an attempt to draw relevant conclusions on the contribution of HGT to tumor evolution.

## Introduction

Generally, it is considered that cancer cells divide by mitosis and do not exchange DNA with each other or with the cells of the tumor microenvironment (1, 2). The vertically inherited mutations across successive generations largely (but not exclusively) determine the adaptive reaction of the cell’s progeny against the existing selective pressure and result in the expansion or contraction of their subclones (1, 2). Recent studies suggested that a non-vertical transmission of DNA may also occur among the community of cancer cells (oncobiota) and between cancer- and microenvironmental genomes (3, 4). This process, namely horizontal gene transfer (HGT), may provide a selective advantage for the recipient cell if the overall effects of the transferred gene are beneficial (5). This phenomenon is considered as a non-cell-autonomous mechanism, in which higher genotype’s adaptive value of the recipient cell is partially linked to the donor cells. HGT may accelerate genomic evolution by allowing a faster adaptation in the group of recipient cells than it would happen by vertical transfer of the same gene (5). This exchange of genetic material occurs among both nearby cells (as paracrine signals) and relatively distant ones (in an endocrine manner) (3, 6). Biologically active cell-free DNA (cfDNA), as a mediator of HGT, can be transported in several forms in the intercellular space (**Box 1**). Here, we particularly focus on the cfDNA-carrying lipid bilayer membrane-enclosed extracellular vesicles (EVs) which can be intercellular mediators of biological and cellular functions. EVs are secreted by most (if not all) cells both under physiological and pathological conditions (14). The cfDNA encapsulation by EVs confers enhanced stability to the transported genomic material (15). Thus, the increased EV secretion of cancer cells (compared to normal ones) and the expanded appearance of specific, clinically relevant mutations found in conveyed DNA allow for the detection and monitoring of tumors using liquid biopsy applications (16–18). However, the evolutionary effect of the released mutant genes inserted into the genome of recipient cells is less known. As we present in **Box 1**, EVs are divided into different subpopulations based on their biogenesis. Given that in most instances there is no direct evidence for the biogenetics route of a given vesicle, an operational classification based on EV sizes can be used. From all EV subcategories, apoptotic cell-derived large EVs (i.e., apoptotic bodies) were first described as mediators of oncogenic HGT (6). Active secretion of the EV-conveyed DNA associated with small EVs (sEVs, **Box 1**) may be particularly important from a clinical perspective, because sEVs from surviving cells may deliver “tried and tested” genes that stimulate fast protective functions against the intense stress factors. Here, we will discuss the main steps of the sEV-DNA-mediated HGT among donor and recipient cells in human cancers, and where it is possible, we compare it to the processes of normal mammalian cells. We particularly focus on the potential biological relevance of sEV-DNA and the controversial issues association with HGT in tumor evolution.

Box 1 **Carriers of cfDNA, EV subcategories, sEVs, exosomes and non-conventionally released vesicles.**

CfDNA as a mediator of HGT, can be present in different forms in the extracellular space, namely as DNA fragments, virtosomes [a complex of DNAs, RNAs, proteins, and lipids (7)], nucleosomes (DNA wrapped around an octamer of histone proteins), or packaged into extracellular vesicles (EVs) (8). The EV nomenclature refers to the EV biogenesis including: i) exosomes (sEVs with endosome/multivesicular body (MVB) origin; ~50-100 nm), ii) microvesicles (originated by direct budding/blebbing from the cell surface; 100-1000 nm) and iii) large EVs including apoptotic bodies (products of apoptotic cell disassembly; 1-5 µm) (9). In most instances, only the diameter of EVs can be determined with certainty. In this review, we follow the MISEV 2018 guidelines which suggest the term sEVs for EVs smaller than 200 nm in diameter, regardless of their origin (10). It follows from the above that the sEV term denotes a group of EVs with heterogeneous origins. Exosomes represent a subset of sEVs which are formed intracellularly by the inward budding of the limiting membrane of endosomes/MVBs (with the intrusion of the cytosolic components). Later, the MVBs fuse with the plasma membrane, so their intraluminal vesicles are released into extracellular space as exosomes. After the uptake, exosomes are known to mediate a wide spectrum of effects on the recipient cell (11).

Recently, unconventional sEV release mechanisms have been hypothesized. In this case, migrasomes and *en bloc* released MVB-like EV clusters could possibly serve as sources of sEVs upon rupture of their limiting membrane (12, 13).

## Release of DNA through sEV secretion

**Figure 1:**
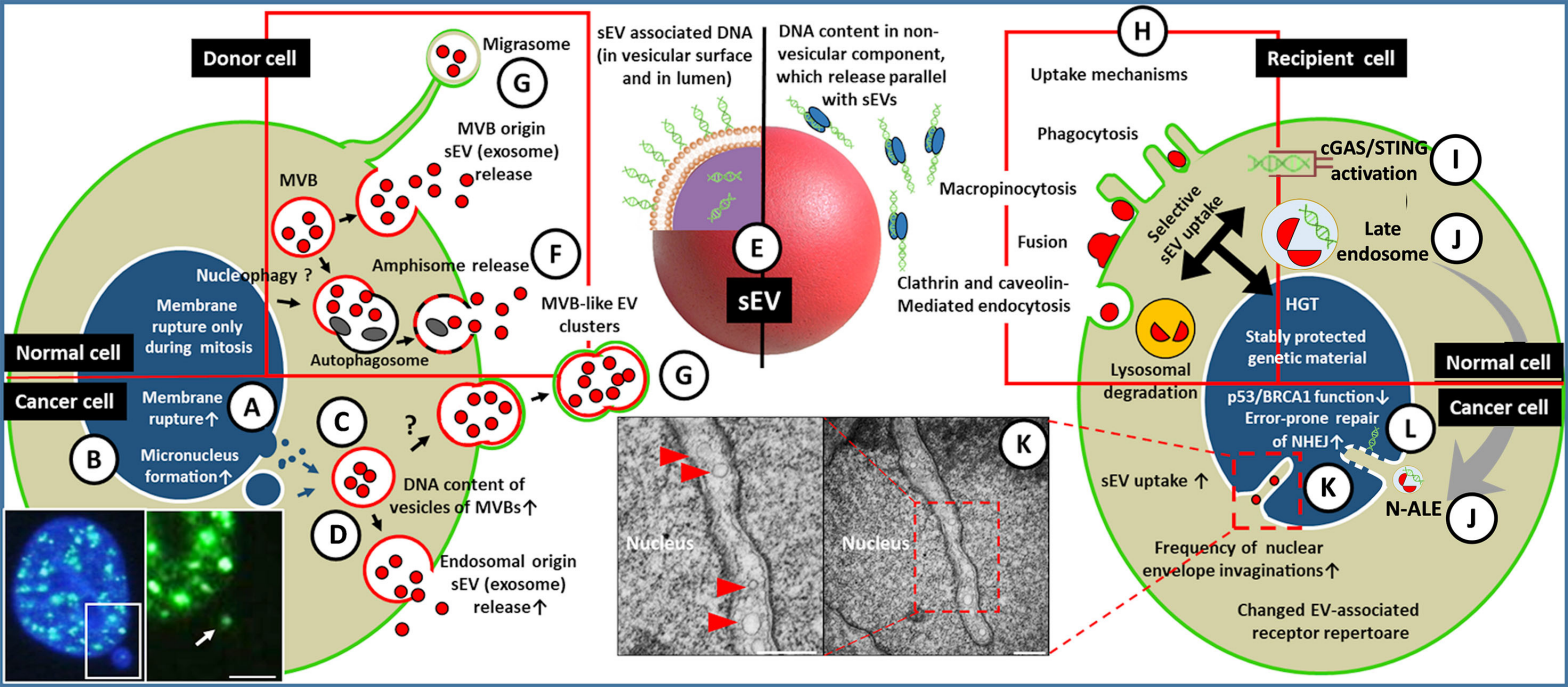
A simplified representation of sEV-mediated HGT among tumor/microenvironmental cells. **(A)** The nuclear gDNA is discharged to the cytoplasmic region of the EV releasing cell (left) through the rupture of the nuclear envelope or by the formation of micronucleus (the fluorescent microscopic image shows a double-strand break (DSB) site in the micronucleus of HT-29 cell [white arrow, γH2AX staining, scale bar: 2 µm)] **(B)**. **(C)** From the cytoplasm or from micronuclei the gDNA translocates into the intraluminal vesicles (future exosomes) of multivesicular bodies. **(D)** Both the gDNA content of the exosomes and their release are increased in tumor cells, especially upon the effect of therapeutic stress. **(E)** The gDNA may be transferred either in the lumen of sEVs and/or on the exofacial EV surface, or independently as a non-vesicular component. The gDNA content of sEVs might depend on their origin, like exocytosis of MVBs **(D)**, amphisomes **(F)**, or sEV discharge from *en bloc* released MVB-like EV clusters or migrasomes **(G)**. The red frame indicates that all listed processes (i.e., migrasome formation, exosome secretion, amphisome exocytosis) may occur in both normal and tumor cells. **(H)** Uptake of the released sEVs by a recipient cell (right) may include receptor-mediated processes (characteristic of both normal and tumor cells, indicated by a red frame). **(I)** In the cytoplasm, the sEVs (or their components) may activate DNA recognition pathways e.g., cGAS/STING. **(J)** The EV-containing late endosomes may reach the invaginations of the nucleus (as nuclear envelope invagination-associated late endosome/N-ALE) where its parts (probably including EV-DNA) may enter the nucleus through nuclear pores. **(K)** Small vesicles (<200 nm) are also detectable in association with the nuclear membrane invaginations (red arrowheads in electron microphotographs of HT29 colorectal cancer cells). The origin of these vesicular structures has not been examined (scale bars: 500 nm). **(L)** The integration of gDNA into the recipient genome may require malfunction of the host DNA repair or onco-suppressor mechanisms (e.g., p53, and BRCA1). Many of the processes presented here have only been described in relation to cancers. Further studies are needed to demonstrate whether these occur in healthy cells.

The majority of cancers are derived from a single ancestral cell by the generation of a diverse successor population with subclonal architecture (1). Cancer also shapes its own microenvironment into a supportive one (19). The developing genetic-, epigenetic- functional- and phenotypic heterogeneity of individual cells provides a remarkable capacity for a population to adapt to challenging environmental conditions during cancer progression and therapy (20, 21). HGT can significantly influence the evolutionary trajectory of a given tumor by spreading genes encoding for molecules which provide advantages for the cells with suboptimal survival, expansion or metastatic capacity. EV-mediated HGT is not a common event among healthy mammalian cells (22). However, the fundamental differences between cancer and normal cells may change the frequency of the EV-mediated HGT. The first alteration in cancer can be the abnormal transport of the genomic DNA (gDNA) from the nucleus to the cytoplasm. Aberrant expression of nuclear membrane components, abnormalities in chromosome segregation, and mechanical forces from the actin cytoskeleton resulting in the rupture of the nuclear envelope are significantly more often observed in cancer than in normal cells, where the disintegration of the nuclear membrane is transient and limited to the mitosis (23). Disruption of the membrane barrier around the chromosomes allows gDNA to be exposed to cytoplasmic locations outside of the nucleus (**Figure 1A**) (23). Another form of delivery of gDNA to the cytoplasm may occur through micronucleus formation (**Figure 1B**), when a chromosome (or part of it) segregates improperly during mitosis and recruits its own nuclear envelope outside of the primary nuclear membrane (24, 25). Other, phenomena which are not studied in association with oncogenic sEV release, such as nucleophagy might also take a part in this process (26). Cytoplasmic or micronucleus-enclosed gDNA can translocate into the intraluminal vesicles of multivesicular bodies (MVBs) (27) (see exosomes in **Box 1** and **Figure 1C**). Although the molecular background of DNA packaging to sEVs remains largely obscure, some interesting details have been described recently. Such a process is the interaction of CD63 exosome-associated tetraspanin with a DNA binding protein (i.e., the presence of CD63-Histone H2B-gDNA complex) which potentially plays a key role in the loading of micronuclear gDNA into sEVs (24). The sEV release is involved in the ablation of potentially harmful or damaged genetic material from the cell (28), suggesting that sEV-mediated gDNA release may be at a baseline level both in normal cells as well as cancer cells to maintain cell homeostasis. However, it is particularly important that this system can adapt quickly to stress (e.g., genotoxic oncotherapies) by increasing micronucleus formation and the concomitant packaging of gDNA into sEVs (sEV-gDNA) (**Figure 1D**) (24). The dynamic adaptation of sEV-mediated DNA release is further supported by the changing quality and/or quantity of transported genetic content (e.g., the proportion of genomic and mitochondrial (mt) DNA, as well as the average size of DNA fragments) upon diverse environmental effects (29, 30).

Considering that the sEVs-conveyed gDNA fragments represent the entire host genome and contain full-length oncogene sequences (31) (**Figure 1E** **)**, they are promising tools for the diagnosis and treatment monitoring of cancer patients by detecting mutations characteristic of the given tumor type (32, 33). However, several recently published studies report conflicting results on the actual DNA transport capacity of sEVs (details in **Box 2**, the described secretion mechanism is indicated in **Figure 1F** **).** As described in **Box 2**, some studies only consider luminal (protected, intravesicular) DNA as a genetic material delivered by sEVs, although DNA can also be associated with the surface of sEVs. Therefore, the DNA transport capacity of sEVs may be underestimated or misinterpreted.

Box 2 **Questions about the ability of sEVs to transport DNA.**

It is generally accepted that sEVs carry higher amounts of double-stranded DNA as compared to the single-stranded one determined by using DNases that differentially recognize and digest the two types of DNA (such as Shrimp dsDNase and S1 nuclease) (16). Besides gDNA, the full mitochondrial genome is also identified in sEVs (34, 35). Furthermore, enhanced amounts of sEV-associated mtDNA and tumor-specific gDNA have been described in the blood of cancer patients (35, 36). This latter property allows sEV-based identification of informative mutations from liquid biopsy applications, supporting the early detection and diagnosis of cancer as well as monitoring the treatment response (15). This established view is questioned by the suggestion that DNA released by cells is localized in DNaseI-sensitive, non-vesicular structures (nucleosomes) released by the exocytosis of amphisomes (37). Although the release of other non-membranous, small (~ 30 nm) nanoparticles (i.e., exomers) has also been described, the authors have detected DNA in sEVs with cell-type-specific relative abundance (38). Furthermore, many earlier studies used DNase treatment for the examination of luminal (protected) DNA [e.g., (22, 31, 35, 39)]. Importantly *Thakur et al.* described that the large (> 2.5 kb) double-stranded DNA fragments bind to the exofacial surface of exosomes, while the size of the luminal DNA fragments ranges from 100 bp to 2.5 kbp (16).

Although DNase treatment has confirmed the presence of luminal DNA content in sEVs in these studies, the question arises whether the DNA removed from the exofacial surface can indeed be classified as an artifact or the surface-associated DNA cargo is a native property of sEVs. This latter possibility was confirmed by an artificial selection pressure induced release of both DNA and DNA-binding proteins on the exofacial surface of sEVs *in vitro* (29). Based on this observation, it should be considered, that although DNase digestion removes potential foreign contaminants of sEVs, it may also eliminate the external, physiological, or pathophysiological DNA cargo from their exofacial vesicular surface.

Conceivably, we propose, that DNA from the microenvironment may also be transferred onto the sEV surface in the extracellular space (as part of a biomolecular corona). Thus, the genetic information carried by an sEV is not necessarily limited to a single EV donor cell.

The DNA cargo has been reported to be present only in a small proportion of sEVs (~10% *in vitro, ~*1% *in vivo*) (16, 24) suggesting that these vesicles are heterogeneous in this respect. This heterogeneity has also been confirmed using high-resolution iodixanol density gradients, discriminating high-density sEV fraction with enriched DNA content and a low-density sEV fraction which carried minor quantities of DNA (40). Because the sEV fraction separated by ultracentrifugation can be derived from multiple cell sources, we need to emphasize the heterogeneity both within sEVs of the same biogenetic origin (e.g., exosome subpopulations) and sEVs released from different subcellular structures (i.e., MVBs, migrasomes, MVB-like EV clusters, **Figure 1G**, **Box 1**).

Probably, the packaging of the genomic nuclear content to sEVs cannot be simplified to a yes/no question. As detailed above, the gDNA content of the cytoplasmic region may be highly dependent on the existence of certain pathophysiological processes in the cell (e.g., nuclear membrane rupture and micronuclei formation) (23–25). It may lead to an increased gDNA content in MVB-originated sEVs (i.e., exosomes) (24, 27). The rapid change in gDNA content of exosomes by existing stress factors (e.g., effects of artificial selection or the pro-inflammatory microenvironment) have been observed (24, 29, 30), however, it is not known how similar stress conditions may regulate the non-MVB-originated sEV release. Presumably, these sEV release pathways may change dynamically upon exposure to various microenvironmental or therapy-induced stress factors.

## Uptake and functions of sEV-gDNA in recipient cells

The tumor can be considered as an ecosystem in which both the cells and the subclonal populations cooperate with each other for acquiring space and resources from the host organism e.g., by sharing molecules with beneficial local and/or systemic effects (41–43). After the sEV-protected gDNA survives the release and is present in the blood or in other body fluids, the recipient cells may internalize it from the extracellular space [the known sEV uptake mechanisms are shown in **Figure 1H**, and were reviewed by McKelvey et al. (44)]. In fact, this process is not trivial, so the selective sEV uptake may be the first barrier to the spread of vesicle-carried genes with oncogenic potential. This selectivity shows the dependency on the type, oncogene status, and receptor repertoire of the recipient cells. Substantial differences in sEV uptake have been observed between normal epithelial cells compared to the tumorigenic variant of them (45), resulting in functional changes in the latter. This study also described robust sEV uptake by fibroblasts (compared to normal epithelial cells), although these fibroblasts lacked proper tumor suppressor mechanisms (45). In the absence of control fibroblasts, it is questionable whether increased sEV uptake was caused by cell-type dependency or other changes, associated with the malfunction of the tumor suppressor mechanisms (see below). The image is further refined by the observation that sEV adhesion to fibroblasts shows integrin receptor dependency resulting in a non-random (i.e., organotropic) metastasis formation (46). On the other hand, the sEV tetraspanin web also plays a primary role in selective target cell binding (i.e., formation of tetraspanin–integrin complexes) (47), which drives attention to the significance of proper donor and recipient cell selection when designing experiments. We have very limited knowledge about the uptake of unconventionally secreted sEVs [such as migrasomes and *en bloc* released MVB-like EV clusters (12, 13)]. Although uptake of migrasomes by recipient cells has been described *in vitro* (12), the details of this process are unknown.

The sEV-DNA uptake may be a robust process as it has been detected in ~16% of sEV-treated fibroblasts (48). The DNA, which enters the cytoplasm does not necessarily reach the nucleus but can activate cytoplasmic DNA recognition receptors (**Figure 1I**). For instance, radiation or chemotherapy-induced, tumor-derived sEV-gDNA triggers antitumor immune response in dendritic cells by activating the cGAS/STING pathway (49, 50). In other cases, sEV-containing late endosomes have been shown to migrate to the invaginations of the nuclear envelope (51), where hypothetically, they can exchange genomic content between the donor sEVs and the recipient cell (**Figure 1J**). However, evidence for such an exchange is still lacking. The EV components may be released from the envelope invagination-associated late endosomes to the narrow space between the endosomal and nuclear membranes and might translocate into the nucleoplasm *via* nuclear pores (51). Ultrastructural studies of our research group detected single, small (<200 nm) vesicles within the nuclear invaginations of cancer cells (**Figure 1K**). However, the origin of these vesicles remains unknown, and obviously, their potential role in HGT should be investigated.

The last step of the oncogenic HGT is the process in which the transferred gDNA integrates into the recipient genome. Some factors have been described in prokaryotes and eukaryotes, which influence the rates of acceptance of the horizontally transferred genes, such as physical/biological properties of the acquired DNA (e.g., length, GC content, codon usage, epigenetic marks, and the complexity of interactions with other genes) as well as the location of genetic integration in the recipient genome (52, 53). However, the last phase of HGT between mammalian cells, especially in sEV-mediated processes has been less examined. Interestingly, genome engineering provides an opportunity for a more in-depth study of this process. The integration of donor DNA sequences at off-target double-strand breaks (DSBs) has been described during CRISPR-Cas9-assisted genome editing, which is known to be caused by an error-prone repair of a non-homologous end joining (NHEJ) pathway (54). The presence of bovine gDNA sequences were detectable in the genome of NIH-3T3 fibroblasts using a medium containing 10% fetal bovine serum. This effect was significantly reduced with the use of exosome-depleted but cfDNA-containing medium, suggesting a primary role of sEVs in HGT (54). Tumor suppressor mechanisms such as the one mediated by p53 may affect the success of genome editing (55, 56). For example, the DSBs can be toxic for human pluripotent stem cells in a p53-dependent manner (55). Similarly, to genome editing, in cancer evolution, the erroneous rejoining of DNA has been shown to also generate genomic changes at DSBs sites (57). This raises the possibility that the capture of exogenous oncogenic sequences at DSB sites might be an evolutionary driving force of tumors. Considering that p53 plays a fundamental role in the fidelity control of NHEJ (58), loss of p53 function can improve tumor cell survival, and in parallel, may create the opportunity for possible HGT. This hypothesis is supported by a pioneering work that showed that p53 dysfunction is required for the incorporation of oncogenes into the recipient genome during apoptotic EV-mediated HGT (6). The fidelity of DNA end-joining was impaired also in the case of *breast cancer gene 1* (*BRCA1*) mutations (**Figure 1L**) (59, 60). The involvement of this tumor suppressor in sEV-mediated HGT is evidenced by the fact that a successful sEV-mediated DNA transfer has been described in *BRCA1*-KO fibroblasts in contrast to wild-type control cells (48).

A successful sEV-mediated HGT can be confirmed *in vitro* by genomic profiling of the transformed cell, detecting integrated gDNA, or its transcription products, phenotypical transformation as well as the new functions associated with the transferred genes. It should not be forgotten that the sEVs transport complex sets of information (61). Thus, the appearance of some new, cancer-associated functions may not only result from the transfer of sEV-gDNA, but also from several other sEV-conveyed regulator molecules. According to the principle of Darwinian selection, the transferred genes can spread in the population, if it carries genetic components that act as drivers associated with the host cell phenotype (5, 6, 45). Consequently, during the study of the long-term effects of sEV-mediated HGT, the tumor evolutionary aspects should also be considered (see below and **Box 3**).

## The potential impact of sEV-mediated HGT in tumor evolution

The tumor-associated sEV signaling, including the delivery of aberrantly released molecule packets (e.g., proteins, lipids, metabolites, coding- and non-coding nucleic acids) influences the evolutionary events of tumors by various, often parallel cellular processes (45, 80). Being the most cancer-specific component of this complex system, here we particularly focused on sEV-delivered gDNA in tumor evolution. Although oncogenic HGT is less known among tumor evolutionary biologists, the *in vitro* and *in vivo* results discussed here clearly indicate that this process is more than a theoretical phenomenon. The presence of clonal heterogeneity in cancer (81) suggests that the HGT-based cooperation among the admixed- or the spatially non-uniformly distributed subclones may be a rare event. Regarding its frequency, it must be emphasized that some phenomena associated with oncogenic HGT can be highly context-dependent (23, 24, 29, 30). Thus, the successful incorporation rate of sEV-mediated HGT may differ greatly depending on the imposed selection pressure. Cellular experiments modeling sEV-gDNA transfer under diverse selective pressures would be clinically relevant for mapping the transfer of resistance mutations between cells of oncobiota. In addition, several experiments listed here focusing on cancer-fibroblast interactions suggested the role of sEV-mediated HGT in adaptive strategies to construct specialized niches. The development of a permissive and subsequently supportive stroma from the tumor suppressor microenvironment is a complex eco-evolutionary process (82), in which the recipient cells may incorporate genetic material from other cells (45, 48). Seemingly, the main requirement for this is that the recipient genome is not stably protected and/or repaired when damaged. This condition is met in carcinoma-associated fibroblasts upon genetic or epigenetic downregulation of *p53* and *BRCA1* [summarized in ref (83)], as well as apoptotic EV-conveyed *HPV16/18 E6* DNA have been shown to impair DNA repair mechanisms. These latter EVs were isolated from cervical cancer and contributed to the disruption of the p53/p21 pathway in primary fibroblasts (84).

By sEV-mediated HGT the recipient cells acquire adaptive benefits which can be manifested in increased proliferation, metastatic capacity, and foci-forming ability, reduced apoptosis, and the potential emergence of HLA-associated immune escape (31, 45, 48). However, these pioneering papers did not investigate HGT under intense therapeutic stress. The importance of therapy in an evolutionary context is highlighted by an observation about apoptotic EV-mediated HGT (6). [Here we note that although the apoptotic EVs arise as typical products of chemo- and radiotherapy, the transfer of full-length (3308 bp) sequences of *H-ras* (one of the examined genes in ref (6)) by sEVs was also described among living cells (31)]. When the incorporated DNA contains an advantageous mutation in the context of the treatment in question, it may become fixed, and it may spread among the recipient offspring cells. Thus, it may contribute to tumor evolution through several generations (6). Accordingly, EV-mediated HGT may greatly affect the sensitivity profile of the cells in residual disease [often undetectable, small population of malignant cells which persist after therapy (85)]. Consequently, its inheritance to the recipient genome may determine the properties of the recurring tumor.

Furthermore, sEVs from the primary tumor may influence critical events of metastasis, such as the preparation of pre-metastatic microenvironment (46) and may induce the formation of potentially metastatic tumor cells at least partly *via* HGT (48). The metastatic spread by genetic material has been known for a long time (86), and it is consistent with Darwin’s pangenetic explanation (87). However, the role of sEVs in this process has only recently been studied and described. During the metastatic cascade, the phenotype of cancer cells shows dynamic changes, including epithelial-to-mesenchymal-, and mesenchymal-to-epithelial transitions (88, 89). The transition of *BRCA1-*KO fibroblasts to carcinoma-like cells (i.e., mesenchymal-to-epithelial transition) could be induced by sEV-gDNA (along with sEV-associated regulators) without preceding epithelial-to-mesenchymal transition (48). This suggests that the metastasic colonization is not exclusively due to the migration of primary tumor cells to metastatic sites [see the “seed to soil model” by Stephen Paget (90)], but it may also involve sEV-induced reprogramming of fibroblasts (48). In connection with this completely new phenomenon, further studies are needed to clarify as to whether carcinoma–like cells behave as tumor cells or as supporting microenvironment cells.

It is important to note that the above-mentioned studies examined the transition of one, or a few selected genes and their short functional effect with potential evolutionary benefits. However, parallel with beneficial genes, neutral or deleterious mutations are also conveyed into the recipient cells by HGT (5). Hypothetically, therefore, the role of oncogenic HGT in cancer may be twofold: transferring deleterious genes may accelerate the irreversible accumulation of mutations which ultimately cause a mutational meltdown. Secondly, it may also increase the genetic diversity required for rapid adaptation by transferring beneficial genes (see **Box 3** for further details).

Box 3 **Horizontal gene transfers to the rescue - Overcoming genomic decay, Muller’s ratchet and metabolic exhaustion in cancer cells**

A cancer cell’s fitness is governed by its own proliferation; thus, the underlying Darwinian dynamics will select for proliferative self-renewal, territorial expansion, migration and invasion properties that procure higher fitness (62, 63). However, the propagation of clonal cancer cells by asexual reproduction exposes them to the emergence and accumulation of recessive mutations (termed ‘‘Muller’s ratchet”). While cancer progression has largely been attributed to selection driving the accumulation of a certain number of somatic mutations, moderately deleterious mutations with no role in cancer (passengers) can accumulate as they largely evade natural selection, and thus negatively alter the cancer evolutionary landscape (64, 65). In the absence of meiotic recombination that would purge deleterious mutations in sexually reproducing organisms, and thus prokaryotes largely rely on horizontal gene transfer to restore and augment genetic diversity (66, 67). While direct evidence is so far lacking for nuclear cancer genomes to rely on HGT to mediate Muller’s ratchet, evidence of capturing host mtDNA to prevent deleterious homoplasmy and loss of mitochondrial function emerges from both human and animal cancer studies (68–70). For example, EVs have been found to harbor and transfer full mitochondrial genomes to cells with impaired metabolism, and thus restore the metabolic activity of breast cancer (34). Mitochondria exchange between leukemic cells and mesenchymal stem cells has also been found to enhance the survival and therapy resistance of leukemia cells (71). In addition, studies show that tumor cells receive mtDNA from other cells of the body in order to maintain optimal cellular respiratory conditions to achieve metastasis (71, 72).

Conquering Muller’s ratchet and maintaining metabolic potential is particularly important for the survival of transmissible cancer cell lines that are able to spread across hosts and hence are being passaged infinite number of times (69). One such transmissible cancer cell lines is the Canine Venereal Tumor (CTVT), a sexually transmitted malignant cell line that affects dogs (73). CTVT is the oldest known living cancer with an estimated age of between 4,000 and 11,000 years (74–77). Since its emergence in Asia (77), CTVT has spread across the globe, infected millions of dogs, and most likely experienced the accumulation and homoplasmy of deleterious mtDNA mutations. To avoid genomic melt-downs and metabolic catastrophes, CTVT has been found to capture and incorporate host mtDNA multiple times, as well as to occasionally employ mtDNA recombination and re-assortment during its evolutionary history (78). Replacement of part of the cancer mtDNA genome with sequence from the host mtDNA has also been observed in another transmissible cancer cell line, the bivalve transmissible neoplasia (BTN) from Chile (79). Whether exosomes have been facilitating HGT in these unique cancer cell lines, remain to be answered and an intriguing research area to follow up.

## Conclusion

Presumably, under physiological conditions, mammalian sEV-mediated HGT may not be extensive, and it has a restricted evolutionary impact. However, we want to emphasize that cancer-associated alterations in DNA repair, sEV secretion and uptake, and functional integration of the transmitted genes might modify the typical range and effect of HGT. Considering that cancer is a special evolutionary system, with its fast-growing, closely spaced large populations of cells that have similar genomes, the effect of rare events is likely to be increased as compared to the physiologic non-tumorous conditions. Mapping of the complex HGT phenomenon and integrating the knowledge reviewed here into our thinking of cancer development and progression may help to better interpret genomic data and allow the development of more precise tumor evolution models.

## Author contributions

GV, BÚ, EIB, TK, SS and ÁK wrote the manuscript. PI, ZT, IT and BM provided the critical revisions. All authors approved the final version of the manuscript for submission and approved it for publication.

## Funding

This work was funded by the NVKP_16-1-2016-0004 grant of the Hungarian National Research, Development and Innovation Office (NKFIH), as well as the Higher Education Institutional Excellence Programme of the Ministry of Human Capacities in Hungary, within the framework of the molecular biology thematic program of Semmelweis University.

## Acknowledgments

We would like to thank István Csabai, Gergely Szöllősi, Alexandra Kalmár, Zoltán Szállási, Barbara Barták, Sára Zsigrai and Norbert Solymosi for their ideas and supports.

## Conflict of interest

The authors declare that the research was conducted in the absence of any commercial or financial relationships that could be construed as a potential conflict of interest.

## Publisher’s note

All claims expressed in this article are solely those of the authors and do not necessarily represent those of their affiliated organizations, or those of the publisher, the editors and the reviewers. Any product that may be evaluated in this article, or claim that may be made by its manufacturer, is not guaranteed or endorsed by the publisher.
